# Urinary incontinence in hospital patients: prevalence and associated factors ^1^

**DOI:** 10.1590/1518-8345.2139.2970

**Published:** 2018-01-08

**Authors:** Jaqueline Betteloni Junqueira, Vera Lúcia Conceição de Gouveia Santos

**Affiliations:** 2 Especialist, Stomal Therapy Especialization, MSc, Escola de Enfermagem, Universidade de São Paulo, São Paulo, SP, Brazil, Nursing, Medic Clinical, Hospital Universitário da Universidade de São Paulo, São Paulo, SP, Brazil.; 3 Post-Doctoral degree, Associate Professor, Departamento de Enfermagem Médico-Cirúrgica, Escola de Enfermagem da Universidade de São Paulo, São Paulo, SP, Brazil

**Keywords:** Urinary Incontinence, Epidemiology, Prevalence, Cross-sectional Studies, Nursing, Hospitalization

## Abstract

**Objectives:**

to analyze the prevalence of urinary incontinence and its associated factors in hospital patients.

**Method:**

this is a cross-sectional epidemiological study whose data were collected using the instruments Sociodemographic and Clinical Data, Characteristics of Urinary Leakage and International Consultation on Incontinence Questionnaire - Short Form. Prevalence was surveyed on a single day for four consecutive months. Data were analyzed using Chi-square test, Fisher’s exact test, Student t-test, Mann-Whitney test and logistic regression (forward stepwise).

**Results:**

the final sample consisted of 319 hospital adults (57.1% female), mean age of 47.9 years (SD=21.1). The prevalence of urinary incontinence was 22.9% (28% in women and 16.1% in men) and the associated factors were: female sex (OR=3.89), age (OR=1.03), asthma (OR=3.66), use of laxatives (OR=3.26), use of diaper during the evaluation (OR=2.75), use of diaper at home (OR=10.29), and use of diaper at some point during the hospital stay (OR=6.74).

**Conclusion:**

the findings of this study differ from those found in the scarce existing literature on the subject in hospital patients. There is a need for previous studies such as this before proposing the implementation of preventive and therapeutic actions during the hospital stay.

## Introduction

The International Continence Society (ICS) defines urinary incontinence (UI) as a “complaint of involuntary leakage of urine” and classifies it as *urge urinary incontinence (UUI)* when involuntary leakage of urine is associated with urgency, as stress *urinary incontinence (SUI)* when there is involuntary leakage of urine during physical effort or physical activity, and as *mixed urinary incontinence (MUI)* when involuntary leakage of urine is associated with urgency, as well as physical exercise, exertion, sneezing and coughing^1^. This is a disorder that negatively impacts the social and sexual relationships, causes psycho-emotional changes and decreased quality of sleep/rest^2^ and its severity has been described as a predictor of quality of life^3^.

International studies indicate an overall prevalence of UI ranging from 8.2% to 26.8% (from 13% to 38.7% in women and from 2.9 to 9.9% in men)^4^ ^-^ ^6^ ^)^ and partial prevalence rates ranging from 1.15% to 6.5% for UUI (from 1.15% to 8.2% in women and from 1.15% to 4.5% in men); from 3.2% to 14.1% for UUI (from 5.8% to 21.2% in women and from 0.49% to 3.9% in men) and from 1.2% to 5.6% for MUI (from 1.26% to 9% in women and from 0.8% to 1.26% in men)^4^ ^-^ ^6^. Among the elderly population, such prevalence reaches 29.4% (from 26.7% to 36.3% in women and from 6.4% to 17% in men), also in the international scenario^7^ ^-^ ^8^.

Population-based studies conducted in Brazil show a prevalence rate of UI ranging from 10.7% to 20.1% in the general population, and its incidence is more frequent among women (from 15.6% to 32.9%) than among men (from 3.7% to 6.2%)^9^ ^-^ ^11^. Among the elderly in the community, this prevalence rate reaches 29.4% (36.3% in women and 17% in men), and can reach up to 41.5% in those over 75 years of age^7^ ^,^ ^11^.

Higher prevalence rates were also found in studies with pregnant women, in which estimates ranged from 10.4 to 71.11%, depending on the trimester of pregnancy, and its incidence was more frequent in the last weeks of pregnancy^12^.

In an older study at national level, a prevalence of UI of 35% was found in 77 adults and elderly patients admitted at the University Hospital of the University of São Paulo (HU-USP)^13^.

The studies analyzed revealed that female sex, advanced age, low level of education, skin color/ethnicity, type 2 Diabetes Mellitus, Systemic Arterial Hypertension (SAH), Cerebrovascular Accident (CVA), obesity, asthma, chronic cough, depression, polypharmacy, smoking, food and water intake, dysuria, Recurrent Urinary Tract Infection (rUTI), parity, menopause, cystocele and functional limitation were the factors most strongly associated with the incidence of UI^7^ ^-^ ^9^ ^,^ ^13^ ^-^ ^14^.

As can be seen, epidemiological studies on UI are predominantly population-based or group-specific, and the literature on hospital patients is scarce. There is a need for previous studies such as this before proposing the implementation of preventive and therapeutic actions, which justifies the importance of this study, whose objective was to analyze the punctual prevalence of UI and the sociodemographic and clinical variables associated with its incidence in hospital patients.

## Method

This is an epidemiological, observational, cross-sectional, analytical and descriptive study. 

This study was carried out at the University Hospital of the University of São Paulo (HU-USP), after being approved by the Research Ethics Committees of the Nursing School of University of São Paulo (EEUSP), and the hospital itself (protocols CAAE 51278715.0.0000.5392 and CAAE 51278715.0.3001.0076, respectively). HU-USP is a secondary hospital, a reference in the western part of the municipality of São Paulo. This hospital serves the USP community (students, professors and employees) and the population of its coverage area (regions of Jaguaré, Vila Sônia, Rio Pequeno and Butantã).

The study population consisted of all adult patients admitted at the HU-USP, in the Units of Medical Clinic, Surgical Clinic, Adult Intensive Care Unit (ICU) and Outpatient Clinic during the data collection period. The average rate of bed occupancy during the data collection period was 86.13%, totaling 410 hospital patients.

The sample calculation was based on a overall prevalence of UI of 27%, according to its epidemiology^4^ ^-^ ^7^ ^,^ ^9^ ^-^ ^11^. An error rate (α) of 5% and 95% Confidence Interval (CI) were considered, resulting in a final sample consisted of 303 subjects. 

In order to achieve the calculated sample size, data were collected four times (point prevalence), on a single day, always on the same day of the month (day 18th), during a period of 12h (from 7 am to 7 pm) in March, April, May and June 2016. 

The study sample consisted of patients who met the following inclusion criteria: be 18 or older, be in a conscious state and aided to answer questions or be accompanied by an intervenor at the time of data collection, consent to participate in the research, not be in use of Indwelling Urethral Catheterization (IUC), not have anuria and not have urinary diversion (nephrostomy, cystostomy, vesicostomy, Bricker’s ureteroileostomy). Sixty-four (15.6%) patients were excluded from the study due to age under 18 (16), mental confusion without the presence of an intervenor (15), unconscious state without the presence of an intervenor (3), refusal to participate (4), presence of IUC (20) and anuria (6). In addition, another 27 patients (6.5%) were not interviewed for various reasons: hospital discharge (11), transfer to the surgical or obstetric center (14), medical examination or medical procedures (1) and transfer to another institution (1). The final sample consisted of 319 patients.

All the patients included in this study were interviewed to collect sociodemographic and clinical data, using a specific instrument aimed at *sociodemographic characterization of the sample* (sex, age, skin color, level of education, years of study, occupation, number of children, family income, marital status and religion); *survey on the clinical variables* (hospital admission diagnosis, SAH, type 2 DM, Heart Failure, asthma, Chronic Obstructive Pulmonary Disease (COPD), CVA, spinal cord injury, Multiple Sclerosis, Alzheimer’s disease, Parkinson’s disease, senile dementia, depression, insomnia, smoking, alcoholism, obesity, diarrhea, gynecological/urological/anorectal surgeries, pelvic or abdominal radiotherapy, functional limitation, idiopathic megacolon, Irritable Bowel Syndrome-IBS/Intestinal Inflammatory Disease, intestinal/rectum neoplasia, hypothyroidism, renal disease, liver disease, hemorrhoidal disease, abscess/infection around the anus, trauma or injury to the rectum/anus, anal fissure, rectocele, cystocele, rectal prolapse, uterine prolapse, menopause, pregnancy, type of delivery, episiotomy, laceration, dysuria, Recurrent UTI, previous indwelling urethral catheterization/ intermittent urethral catheterization/ rectal catheterization, use of medications (diuretics, anticholinergics, opioids, hypotensive, laxative, antibiotics, etc.), use of diaper at home, during the hospital stay and during the evaluation, and *identification of the presence of IU*.

The incidence of UI was considered as involuntary leakage of urine. Among those considered as incontinent, two instruments for the characterization of urinary leakage were applied: *International Consultation on Incontinence Questionnaire - Short Form* *- ICIQ-SF* and Characteristics of Urinary Leakage. The first instrument was culturally adapted and validated for Brazil in 2004^15^. It consists of six questions, the first two ones refer to demographic data (age and sex), and the next three ones refer to the frequency and amount of urinary leakage and how much it affects daily life. These questions have partial scores that result in a final severity score when they are added. The last question aims to characterize the situation in which urinary leakage occurs. This instrument has no cut-off point for stratifying the severity of urinary leakage; however, its scores are directly proportional to the magnitude of urinary incontinence. As for the second instrument, developed by Brazilians^13^, it consists of 13 questions that were designed to characterize urinary leakage, including duration, conditions, frequency and amount of urinary leakage and the use and frequency of changes of the urinary incontinence control devices. This instrument has no partial or final scores and the understanding of the magnitude of the severity of UI depends on the interpretation of the professional who applies it. Although it has not been formally validated, its validation has occurred through studies in which it has already been used^9^ ^-^ ^10^. Data collection was supplemented by consulting the medical records and by means of physical examination. Physical examination consisted of a rapid check of the presence or not of IUC or urinary diversion, which are used as criteria for the selection of patients.

The data collected were coded and entered into a spreadsheet of the MS Office Excel®, version 2007, for building up the database. Subsequently, they were transported to the SPSS software, version 22.0, for performing the analyses. In this study, it was used point prevalence, which is the proportion of individuals who have a clinical condition at a certain point of time. Two statistical tests were used for the study of categorical independent variables: chi-square test and Fisher’s test. Student’s t-test and Mann-Whitney test were used to measure the association between numerical variables. In addition, multivariate analysis was performed using logistic regression (*forward stepwise*), to identify the variables associated with the *UI* outcome. For all statistical analyzes of the study, statistical significance was set at 5% (p<0.05). However, for the inclusion of independent variables in the regression model, the level of significance was set at 10% (p<0.1). The Confidence Interval was set at 95%.

## Results

According to Table 1, the sample consisted predominantly of women (182/57.1%), mean age of 47.9 years (SD=21.1), white skin color (147/46.1%), 8.4 years of study on average (SD=4.6), married (169/53%), with paid jobs and formally employed (86/27%). According to Table 2, among the interviewees, 116 (36.4%) patients reported having SAH, 71 (22.2%) had DM, 64 (20%) had functional limitation, 57 (17.9%) had recurrent UTI and 57 17.9%) were smokers. The mean length of hospital stay was 8.4 days (SD=13.5).

**Table 1:** Sociodemographic characteristics of the sample (N=319). São Paulo, SP, Brazil, 2016

Variables	Mean	Median	SD*	Minimum	Maximum
Age	47.9	49	21.1	18	103
Years of study	8.4	9	4.6	0	24
				N^†^	%
Sex					
Female				182	57.1
Male				137	42.9
Skin color					
White				147	46.1
Black				52	16.3
Brown				112	35.1
Yellow				8	2.5
Marital status					
Single				93	29.2
Widower				35	11.0
Common law/married				169	53.0
Divorced				22	6.9
Employment status					
Formally employed				86	27
Informally employed				6	1.9
Self-employed				42	13.2
Casual labor				20	6.3
Retired				81	25.4
Pensioner				13	4
Unemployed				67	21
Work leave				4	1.2

*Standard deviation; †Absolute number

**Table 2:** Clinical characteristics of the sample (N=319). São Paulo, SP, Brazil, 2016

**Variables**	**Mean**	**Median**	SD*	**Minimum**	**Maximum**
**Length of hospital stay**	**8.4**	**13.5**	**3**	**1**	**113**
				**N** ^**†**^	**%**
**Clinical history**					
*Systemic Arterial Hypertension*				**116**	**36.4**
*Diabetes Mellitus*				**71**	**22.2**
*Asthma*				**20**	**6.2**
*Chronic Obstructive Pulmonary Disease*				**23**	**7.21**
*Heart Failure*				**51**	**16**
*Recurrent Urinary Tract Infection*				**57**	**17.9**
*Smoking*				**57**	**17.9**
*Alcoholism*				**19**	**5.9**
*Functional limitation*				**64**	**20**
**Use of medications**					
*Use of diuretics*				**51**	**16**
*Use of laxatives*				**22**	**6.9**

*Standard deviation; †Absolute number

The overall prevalence rate of UI was 22.9% (73), with 28% (51) in women and 16.1% (22) in men.

Among the demographic variables, those showing statistically significant differences between the groups with and without UI were: age (p<0.001), sex (p=0.012), years of study (p<0.001) and level of education (p<0.001). 

Among the clinical variables, those showing statistically significant differences between the groups with and without UI were: SAH (p=0.001), DM (p<0.001), HF (p=0.002), Alzheimer’s disease (p=0.012), asthma (p=0.001), COPD (p=0.003), smoking (p=0.030), functional limitation (p=0.000), hemorrhoidal disease (p=0.04), recurrent UTI (p=0.006), anorectal surgery (p=0.005), use of diaper (p<0.001), use of diuretics (p=0.001) or laxatives (p=0.002). Among women, the variables that showed statistically significant differences were: cystocele (p=0.000), menopause (p<0.001), number of pregnancies (p=0.008), number of deliveries (0.005), natural delivery (vaginal delivery with or without application of forceps) (p=0.001) and vaginal delivery (without application of forceps) (p=0.001). Sexual impotence was significantly more frequent in incontinent men (p<0.001), compared with those without UI. 

As show in Table 3, according to the logistic regression model, the variables that remained associated with UI were: female sex (OR=3.89, 95%CI: 1.8-7.9), age (OR=1.03, 95%CI: 1.01-1.05), asthma (OR=3.66, 95%CI: 1.3-10.2), use of laxatives (OR=3.26, 95%CI: 1.0-9.8), use of diaper during the evaluation (OR=2.75, 95%CI: 1.0-6.9), use of diaper at home (OR=10.29, 95%CI: 1.8-57.6) and use of diaper at some point during the hospital stay (OR=6.74, 95%CI: 0.4-91.8). According to the logistic regression model, women are 3.9 times more likely to have UI; for each year of age, the chance of having UI increases by 3.6%; to have asthma increases by 3.7 times the chances of having UI; the use of laxatives increases these chances by 3.3 times. The use of diapers by the patient during the evaluation increased by 2.7 times the chances of having UI, whereas the use of diapers at home increased the chances by 10.3 times; and the use diapers at some point during the hospital stay increased these chances by 6.7 times.

**Table 3:** Variables associated with the incidence of urinary incontinence (N=319). São Paulo, SP, Brazil, 2016

Variables	P value	OR*	95%CI^†^	
			Lower	Upper
Sex (female)	0.000	3.896	1.899	7.991
Age	0.000	1.036	1.019	1.054
Asthma	0.014	3.660	1.302	10.290
Use of laxatives	0.035	3.262	1.085	9.811
Use of diaper during the evaluation	0.031	2.752	1.096	6.908
Use of diaper at home	0.008	10.293	1.839	57.606
Prior use of diaper during the hospital stay	0.152	6.749	0.496	91.834

*Exposure Odds or Odds Ratio; †95% Confidence Interval for Odds Ratio

For the application of the logistic regression model to evaluate the association between variables and UI in women, age and asthma were repeated, together with the number of deliveries and DM, as shown in Table 4.

**Table 4:** Variables associated with the incidence of urinary incontinence in women (N=182). São Paulo, SP, Brazil, 2016

Variables	P value	OR*	95%CI^†^	
			Lower	Upper
Age	0.000	1.037	1.017	1.056
DM†	0.041	2.596	1.039	6.489
Asthma	0.010	4.921	1.460	16.588
Number of Deliveries	0.008	1.273	1.064	1.522

*Exposure Odds or Odds Ratio; ^†^95% Confidence Interval for Odds Ratio; †DM - Diabetes Mellitus

Among those people with UI, 24 (32.9%) had it for more than six years, 15 (20.5%) for less than one year and 15 (20.5%) between one and three years; eight (11%) reported the onset of the incontinence after their hospital stay. Regarding the frequency of urinary leakages, they occurred once a week or less in 25 (21.9%) patients and several times a day in 22 (30.1%) patients. Urinary leakage occurred as small amounts in 42 (57.5%) patients and as large amounts in 20 (27.4%) patients. Patients reported that the situations in which urinary leakage occurred more frequently were when coughing or sneezing in 38 (52.1%) patients, followed by its occurrence when the patients were sleeping, 27 (37%). Amongst the incontinents, 21 (28.8%) patients reported that only “sometimes” it was possible to reach the bathroom in time; 53 (72.6%) patients reported waking up at night when they realized that they needed to urinate; 69 (94.5%) patients had no urinary leakage during sexual intercourse. Regarding the use of resources to contain urinary leakage, 52.1% (38) reported using some type of resource, 52.6% (21) of them used diapers, and the other ones reported the use of sanitary napkins. These resources were replaced three times a day in 22 (59.2%) incontinents. When asked about changes in daily life due to urinary leakage, 33.8% (23) of the patients stated that these changes occurred, mainly associated with situations as going out (18/26.5%), in the leisure time (8/11.8%) and during sleep (8/11.8%). Most (56/81.2%) believe that the UI has not affected their relationships. For 24 (35.8%) patients, urinary leakage was considered as a serious problem. 

Amongst the patients with UI prior to the hospital stay, only 16.7% of the patients reported having sought professional help to deal with this problem. 

## Discussion

The prevalence of UI found among hospital patients was 22.9% (28% in women and 16.1% in men), which is much lower than that found in a study carried out in the same hospital, with 77 adults and elderly patients, 35%^13^. No other international study on the prevalence of UI in a hospital population was found, except a Spanish study carried out in 2015 with 924 elderly patients hospitalized in Zaragoza, in which there was a global prevalence of 80% (84.76% in women and 73.9% in men). This study showed that UI was among the ten most common health problems in both sexes, with a prevalence higher than that of widely distributed comorbidities such as SAH and type 2 DM^16^. The prevalence found in this study is similar to that found in national and international studies carried out with the general population, in which the prevalence of IU ranges from 8.2% to 27% (from 13% to 38.7% in women and from 2.9% to 11% in men)^4^ ^-^ ^7^ ^,^ ^9^ ^-^ ^11^.

In a population-based study carried out with 1,705 elderly inhabitants in the municipality of Florianópolis, in the state of Santa Catarina, the prevalence of UI was 29.4% (36.3% in women and 17.0% in men), reaching 41.5% among elderly over 75 years^7^. In another study carried out with older people from seven countries in Latin America and the Caribbean, including Brazil, the self-reported prevalence of UI in the municipality of São Paulo was 11.8% among men and 26.2% among women^10^.

In the present study, female sex, advanced age, asthma, use of laxatives, use of diapers at home, use of diapers at some point during the hospital stay or during the evaluation were associated with the occurrence of UI, according to the logistic regression model. A study carried out with individuals admitted in the same hospital showed a statistically significant correlation between UI in hospital individuals and other factors, such as: dysuria, urinary tract infections, length of hospital stay and male sex^13^.

Despite the popular belief and the large number of studies carried out with elderly patients, UI is not an inherent alteration in the aging process. In fact, its incidence increases proportionately with age and can be considered as a geriatric syndrome^8^ ^,^ ^7^ ^,^ ^17^ ^-^ ^18^. Aging leads to cognitive changes related to body coordination and mobility, as well as the emergence of associated diseases such as neurodegenerative diseases, and these factors contribute to the incidence of UI. 

Unlike the age factor, the association between UI and the use of laxatives is still poorly studied and no other study was found in the national and international literature to corroborate this association. The use of laxatives probably emerged as a factor associated with UI due to the concomitant incidence of UI and constipation in the same individual. Another hypothesis is that due to diarrhea, the individual has a greater difficulty in containing the leakage of urine during evacuation. 

The association between asthma and leakage of urine has been frequently demonstrated by other epidemiological studies^7^ ^,^ ^14^. Respiratory problems lead to chronic coughing or frequent sneezing, which cause repeated increase in intra-abdominal pressure and a consequent pelvic floor overload. A study conducted in 2015 showed that individuals who reported having bronchitis or asthma were 38% more likely to have UI than those who did not have these diseases^7^.

The use of diaper among the factors associated with UI leads to the formulation of some hypotheses about the relationship between these variables. The previous use of diaper at home clearly shows the existence of collinearity between the variables. Therefore, it is naturally expected that those who have long-standing incontinence will wear diapers and vice versa. However, the use of diapers during the evaluation or at some point during the hospital stay raises doubts about the induction of the loss of control of urinary continence due to the use of diapers in previously continent patients. It is known that the use of diapers during the hospital stay may facilitate the care of elderly patients, avoiding efforts to remove them from the bed and take them to the bathroom. In addition, diapers are commonly used during the hospital stay, especially at night, to avoid the risk of falls in elderly patients. An international study showed that the excessive use of diapers in continents patients during the hospital stay was the second most frequent cause of iatrogenesis, that is, it induced UI^19^.

Women are generally the population most affected by UI, and aging is an aggravating factor for the incidence of UI. The incidence of UI in men and women happens in a systematically different way, occurring predominantly in females^4^ ^,^ ^6^ ^-^ ^8^. This association may be due to estrogen deficiency and parity, among other factors^20^ ^-^ ^21^.

Estrogen plays an important role in the pelvic support mechanism, controlling the synthesis and degradation of collagen^20^. In addition, the tissues of the lower urinary tract are sensitive to estrogen, which influences the increase in urethral resistance, the frequency and amplitude of the contractions of the detrusor muscle, and consequently, the sensory threshold of the bladder^21^. For these reasons, the reduction of this hormone after menopause is a possible etiologic factor for pelvic floor disorders, including urinary incontinence^14^.

In this study, there were statistically significant differences between the groups of women with different number of pregnancies and different types of deliveries in relation to the incidence of UI. Among the results, it is worth mentioning the increase in the chances (27.3%) of the woman becoming incontinent at each delivery, regardless of the type of delivery. 

Discussions about the association between the type of delivery and the occurrence of UI are still contradictory, however, the most recent publications suggest that cesarean delivery is a protective factor for UI^22^ ^-^ ^23^. In a study carried out with women who have recently given birth, the frequency of postpartum SUI was significantly higher in vaginal delivery when compared to cesarean delivery after one month (p<0.001), six months (p<0.001) and 12 months (p<0.001)^22^. In a systematic review^23^ that included 16 studies comparing the risk of SUI and UUI between vaginal delivery and cesarean delivery, it was found, except for one study, that there is an increased risk of SUI and UUI after vaginal delivery in comparison with cesarean delivery (OR=1.85 [95%CI: 1.56-2.19]).

In the men group, no variable associated with UI has emerged. According to the previously mentioned study, with hospital individuals, statistically significant correlations were found between the prevalence of UI in men and the increase in age^13^. In other epidemiological studies, radical prostatectomy has been shown as an important risk factor for IU, especially in the immediate postoperative period, with self-reported prevalence rates ranging from 20% to 57% one year after surgery, depending on the surgical method used^24^.

It is important to note that in eight incontinent patients (11%), the problem arose during their hospital stay. During the hospital stay, patients are subject to loss of urinary control due to deterioration of their health status, decreased functionality and frequent decrease in their level of consciousness caused by the disease itself or by sedation. In elderly patients, the incidence of UI during the hospital stay is quite common. At this age, functional alterations are justified by their low functional reserve, which makes them more vulnerable to dysfunctions in situations of organic stress. Among the interviewees who had UI, most of them usually wake up to go to the bathroom when they need to urinate, but only a third of them are able to reach the bathroom in time. In a population study at national level^10^ these rates were higher, 93% woke up in the middle of the night to urinate, and 56.3% did not keep their continence until reaching the bathroom. UUI is common in elderly and is associated with an increased risk of falls and consequent fractures^25^. This risk is even greater at night, usually when the caregiver is sleeping. 

In addition, only 16.7% of patients reported having sought professional help to deal with this problem. This can be explained by the belief that urinary incontinence is an expected phenomenon during aging, which may imply some resilience from those affected with regard to the problem and the development of adaptive actions^18^.

The prevalence of UI found in this study, as well as its associated factors, corroborate the findings of the national and international literature on UI in the general population. However, there is still a gap in knowledge on the occurrence of this health condition in hospital patients. Despite its contribution to minimizing this gap, it is recognized that this study is restricted to only one hospital and presents the limitations of a cross-sectional design study. Thus, this study encourages new studies that will include other hospital institutions, as well as longitudinal data collection, making it possible, mainly, the confirmation of the variables associated with the incidence of UI in this clientele.

## Conclusion

In this study, carried out with 319 hospital patients, it was concluded that the overall prevalence of UI was 22.9%, 28% in women and 16.1% in men. The following variables were associated with UI: female sex (OR=3.89, 95%CI: 1.899-7.991), age (OR=1.03, 95%CI: 1.019-1.054), asthma (OR=3.66, 95%CI: 1.302-10.290), use of laxatives (OR=3.26, 95%CI: 1.085-9.811), use of diapers during the evaluation (OR=2.75, 95%CI: 1.096-6.908), use of diapers at home (OR=10.29, 95%CI: 1.839-57.606) and use of diapers at some point during the hospital stay (OR=6.74, 95%CI: 0.496-91.834).
